# Superior Cluneal Nerve Entrapment Syndrome: Thought to Be Spondylolysis

**DOI:** 10.5435/JAAOSGlobal-D-23-00091

**Published:** 2023-10-18

**Authors:** Tiffany Ruan, Alvin C. Jones

**Affiliations:** From the College of Osteopathic Medicine, Kansas City University, Kansas City, MO (Ms. Ruan), and the Division of Orthopaedic Surgery, Cincinnati Children's Hospital Medical Center, Cincinnati, OH (Dr. Jones).

## Abstract

A rare but typically overlooked diagnosis in the orthopaedic surgery community is superior cluneal nerve (SCN) entrapment syndrome. The cluneal nerves function as purely sensory fibers, and the SCNs provide cutaneous innervation to the posterior parasacral, gluteal, and posterolateral thigh regions. When irritated, this syndrome can cause acute and chronic lower back pain and lower extremity symptoms. A 14-year-old adolescent girl presented to the clinic for an evaluation of pain in the right side of her lower back. The patient's physical examination showed tenderness to palpation on the right posterior iliac crest seven centimeters from the midline. Her neurologic examination demonstrated normal deep tendon reflexes, muscle strength, and sensation in the L2-S1 dermatomal distribution. Although imaging showed evidence of a left L5 spondylolysis, she responded positively to a steroid injection over the posterior iliac crest but negatively to one over the L5 pars defect. She later underwent a right SCN decompression surgery. After the procedure, she reported at least 90% improvement in her pain and rated it as a one in severity, on a scale of 0 to 10. Research regarding SCN entrapment syndrome has increased in the past several years. However, most of these studies are limited to the adult population. Therefore, more reports highlighting the potential for this syndrome in adolescents are needed as well.

Chronic low back pain has historically been deemed a diagnosis affecting the adult population. Recently, adult studies have been exploring the effect superior cluneal nerve (SCN) entrapment syndrome has on chronic back pain. However, research is lacking regarding this diagnosis in adolescents although chronic low back pain is very common amongst adolescents.^1,2^ Superior cluneal nerve entrapment syndrome is overlooked as a potential diagnosis because of the lack of awareness.

Symptoms of SCN syndrome include lower back pain at rest, intermittent lower back pain that involves the iliac crest and buttocks, pain induced by standing and walking, and lower back pain that is improved by a nerve block.^3^ However, unlike a central motor nerve injury or nerve root radiculopathy, patients lacked abnormal deep tendon reflexes, muscle weakness, or abnormal Babinski reflex.^4^ In addition, there was also a lack of tenderness over the vertebrae or costovertebral angles.

The SCNs originate from the dorsal rami of primarily the upper spinal nerves from the T12-L5 nerve roots^5^ (Figure 1). The nerves then cross the iliac spine to innervate the skin and subcutaneous tissue over the gluteal region. The SCNs function as purely sensory fibers, and the superior cluneal branches provide cutaneous innervation to the gluteal and posterior parasacral regions^6^ (Figure 2). When irritated, the nerves can cause acute and chronic lower back pain and lower extremity symptoms described as pseudosciatica.

**Figure 1:**
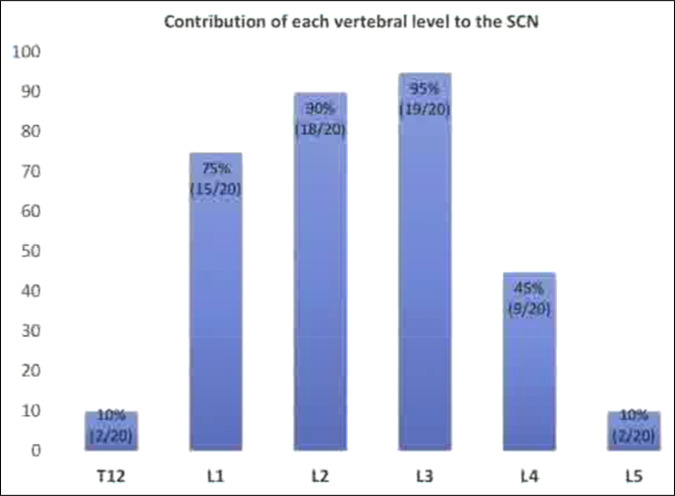
Graph showing dermatomal distribution of the SCNs from T12 to L5.^14^

**Figure 2:**
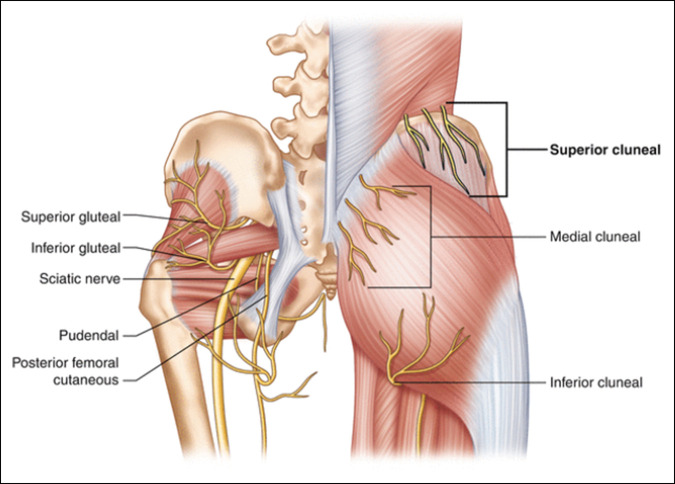
Image showing the anatomical distribution of SCNs.^16^

Although considered a rare disorder, anatomic studies on the SCN reveal evidence of entrapment in about 1.8 to 13% of cadaveric specimens.^7-9^ In addition, studies have attested to the effectiveness of surgical treatments. In one study, releasing the compressed branches of the SCN markedly improved Roland-Morris Disability Questionnaire scores for all 34 patients treated for this diagnosis.^10^

Surgical treatment involves making an incision over the posterior iliac crest about seven centimeters from midline, dissecting down to the fascia, and opening the fascia surrounding the branches as they cross over the iliac crest.^4^ Surgical pathology of SCN entrapment syndrome reveals enlarged nerve branches with decreased myelinated fiber density, increased thinly myelinated fibers, perineural thickening, subperineural edema, and Renaut bodies.^11^

Although awareness is increasing in the adult population, little attention has been paid to this pain disorder in adolescents. We present here a case study of an adolescent with chronic back pain thought to be caused by an L5 spondylolysis yet was subsequently diagnosed and treated for SCN entrapment syndrome.

## Patient Information

**Figure 3:**
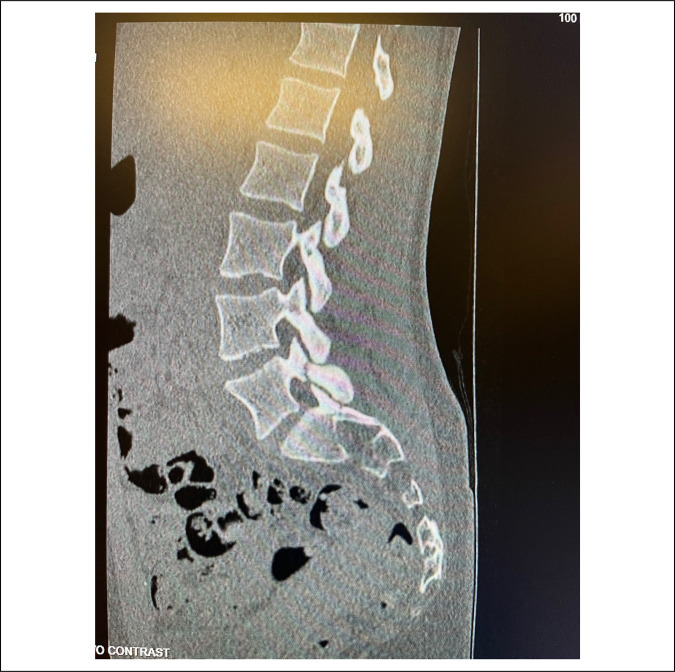
Computerized tomography scan showing pars fracture at the level of L5.

**Figure 4:**
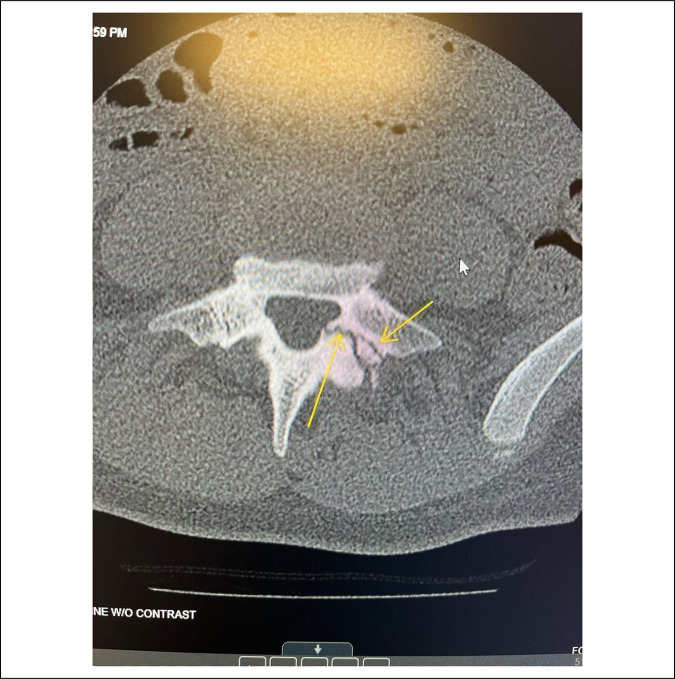
CT scan showing pars fracture at the level of L5 on the left.

A 14-year-old Caucasian adolescent girl presented to our institution's pediatric spine clinic for evaluation of chronic right lower back pain. According to her, the pain began a year ago but was exacerbated after a recent fall onto her right side. Three days before her arrival at the clinic, she presented to the emergency department with extreme pain in her right lower back. She described the pain as constant, aching, stabbing, and sharp. The flare-up in symptoms was present for over 2 weeks, and the severity was rated 6 of 10. She also stated that the pain radiated into her right buttock and right hip. It was aggravated with increased physical activity, standing for long periods, hyperextending her back, lying flat, and bending over. Conversely, it somewhat relieved with decreased physical activity. Previously, she unsuccessfully tried to manage the pain with chiropractic care, NSAIDs, lidocaine patches, rest, heat, and ice. Her mother reported that after her fall, she took her daughter to the emergency department, where a lumbar CT scan revealed a left-sided L5 spondylolysis (Figures 3 and 4).

## Clinical Findings

On examination, she had tenderness to palpation over the right posterior iliac crest about seven centimeters from midline. Her range of motion was limited because of pain with lumbar hyperextension and cross-body forward flexion toward the left. Her neurologic examination was normal, showing normal deep tendon reflexes in the patella and achilles and normal motor strength bilaterally in the extensor hallucis longus, tibialis anterior, gastrocnemius-soleus complex, quadriceps, and hip flexors. In addition, her sensation was also intact to light touch in the L2-S1 dermatomal distribution.

## Diagnostic Assessment

Although the patient's imaging showed a left L5 pars defect, her clinical signs did not match the imaging. Her spondylolysis was located on the left, but her pain was on the right. In addition, the patient's pain was much more lateral on her back that would be expected with a spondylolysis. Furthermore, the radiating pain to the buttock and hip was suspicious for some form of neuropathy.

**Figure 5:**
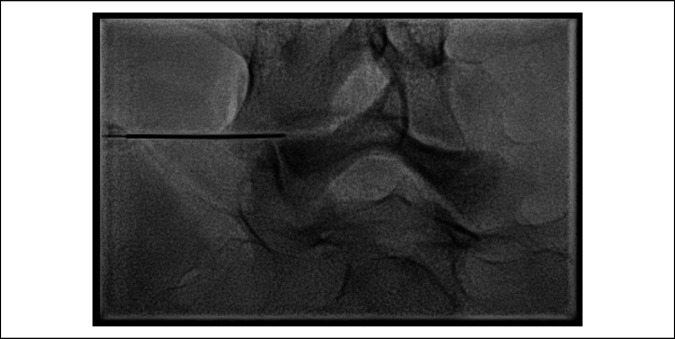
Radiograph showing fluoroscopic-guided injection performed by interventional radiology at L5 on the left while the patient is lying prone on her left to potentially relieve pain caused by the left L5 pars defect.^17^

To further investigate the cause of her pain, the patient received a local injection of 1% lidocaine and 40 milligram depo-Medrol over the right iliac crest, seven centimeters to the right of midline. Six days after the steroid injection, her mother called reporting that the patient was still having notable pain in her right lower back. Therefore, the decision was made to explore the possibility that the pain was from the pars defect, and an order for steroid injection by interventional radiology over the left L5 pars defect was placed. She underwent this 3 weeks after the initial clinic visit (Figure 5). Three days after the L5 pars defect injection, her mother called stating that the patient's pain was worse. Finally, when the patient and her mother returned to the clinic, the timeline of her pain response was clarified for each steroid injection. Her mother reported that for the initial injection, the day after she called, her daughter's pain symptoms actually started to improve. Nevertheless, the mother wanted her daughter to undergo the second injection to see if it would also help, but it did not help at all. At the time of her follow-up clinic appointment, the benefits of the initial injection had worn off. Based on her response to the initial injection, the diagnosis of a right SCN entrapment syndrome was confirmed.

## Therapeutic Intervention

The option of decompression was discussed with the patient and her mother as being more likely to give her long-term relief, and they agreed to undergo a right SCN decompression surgery.

The surgery was performed through a five-centimeter incision over the right posterior iliac crest. Mainly blunt dissection through the subcutaneous fat layer was performed with a cobb elevator. Monopolar cautery was also used sparingly to obtain hemostasis. As the iliac crest was approached, bipolar cautery was used for hemostasis, and the cobb elevator was used to clear the fatty tissues off the fascia. A Beaver blade scalpel and metzenbaum scissors were used to open the fascia along the iliac crest about four centimeters from the posterior superior iliac spine. Several branches of the right SCN were found and appeared edematous. After releasing the fascia surrounding the branches, the wound was irrigated with normal saline, and a layered closure of the wound was performed.

## Follow-up and Outcome

One month after the decompression surgery, the patient presented to the clinic for follow-up. She reported at least 90% improvement in her previous pain and rated it 1 of 10 at its worst. She also reported that her current pain was mostly incisional and only presented when she accidently bumped it on a hard surface. She denied tenderness to palpation in the back or peri-incisional tenderness to palpation. In addition, the patient denied pain with hyperextension or cross-body flexion. No additional imaging was taken after the surgery.

## Discussion

Chronic lower back pain is one of the most common diagnoses in the adult and pediatric populations across the world. More than 200 billion dollars are spent annually for chronic back pain management.^12^ Low back pain due to SCN entrapment syndrome is commonly undiagnosed because of lack of awareness about this disorder. It can be misdiagnosed for another disorder because the clinical symptoms and physical examination findings present similarly to other common lower back disorders, such as spondylolysis or lumbar disk herniation.^13^ However, the radiating pain from SCN entrapment syndrome can frequently be distinguished from a disk herniation by its sensory distribution. The SCN innervation is most commonly in the L1-L3 dermatomal distribution, which typically presents at or above the knee.^14^ There is also a typically negative straight leg raise and no associated muscle weakness. The pain from spondylolysis, on the other hand, will be in the midline and correlated anatomically with the radiographic location of the spondylolysis.^15^

This case study highlights the need for more discussion on this topic amongst orthopaedic surgeons evaluating and treating adolescent patients with chronic low back pain. It is also important for family physicians and pediatricians to be aware of this clinical diagnosis to correctly refer them for further management and treatment. As more research focuses on SCN entrapment syndrome in adolescents, this will ultimately reduce the likelihood of misdiagnosis. Additional research is needed to learn the long-term outcomes and complications of these treatments and to find nonsurgical alternatives to treating this diagnosis.
